# Local recurrence of robot-assisted total mesorectal excision: a multicentre cohort study evaluating the initial cases

**DOI:** 10.1007/s00384-022-04199-3

**Published:** 2022-06-16

**Authors:** T. A. Burghgraef, R. M. P. H. Crolla, M. Fahim, G.P. van der Schelling, A. B. Smits, L. P. S. Stassen, J. Melenhorst, P. M. Verheijen, E. C. J. Consten

**Affiliations:** 1 Department of Surgery, University Medical Centre Groningen, Hanzeplein 1, 9713 GZ Groningen, the Netherlands; 2 Department of Surgery, Meander Medical Centre, Maatweg 3, 3813 TZ Amersfoort, the Netherlands; 3 Department of Surgery, Amphia Hospital, Breda, the Netherlands; 4 Department of Surgery, St Antonius Hospital, Nieuwegein, the Netherlands; 5 Department of Surgery, Maastricht University Medical Centre, Maastricht, the Netherlands

**Keywords:** Robot-assisted surgery, Rectal cancer, Total mesorectal excision, Local recurrence

## Abstract

**Purpose:**

Evidence regarding local recurrence rates in the initial cases after implementation of robot-assisted total mesorectal excision is limited. This study aims to describe local recurrence rates in four large Dutch centres during their initial cases.

**Methods:**

Four large Dutch centres started with the implementation of robot-assisted total mesorectal excision in respectively 2011, 2012, 2015, and 2016. Patients who underwent robot-assisted total mesorectal excision with curative intent in an elective setting for rectal carcinoma defined according to the sigmoid take-off were included. Overall survival, disease-free survival, systemic recurrence, and local recurrence were assessed at 3 years postoperatively. Subsequently, outcomes between the initial 10 cases, cases 11–40, and the subsequent cases per surgeon were compared using Cox regression analysis.

**Results:**

In total, 531 patients were included. Median follow-up time was 32 months (IQR: 19–50]. During the initial 10 cases, overall survival was 89.5%, disease-free survival was 73.1%, and local recurrence was 4.9%. During cases 11–40, this was 87.7%, 74.1%, and 6.6% respectively. Multivariable Cox regression did not reveal differences in local recurrence between the different case groups.

**Conclusion:**

Local recurrence rate during the initial phases of implantation of robot-assisted total mesorectal procedures is low. Implementation of the robot-assisted technique can safely be performed, without additional cases of local recurrence during the initial cases, if performed by surgeons experienced in laparoscopic rectal cancer surgery.

## Introduction

The primary surgical treatment of rectal carcinoma is total mesorectal excision (TME) [1]. The introduction of total mesorectal excision caused a reduction in local recurrence, although systemic recurrence rate remained stable [1]. Subsequently, the introduction of laparoscopic TME (L-TME) caused an improvement of short-term outcomes such as length of stay, but did not improve oncological outcomes [2–6].

Over the last two decades, a new minimal invasive technique has been introduced: robot-assisted TME (R-TME). This technique has been developed to overcome difficulties of laparoscopic surgery, which is especially seen in bulky, low rectal tumours of patients with a small pelvis and a high BMI [7]. It is suggested that R-TME might lead to lower rates of involved circumferential resection margin (CRM) and of incomplete TME specimen, due to increased visibility and precision during the surgical procedure [8, 9]. Up to now no clear benefits regarding post-operative morbidity have been shown [9–11]. However, in patients operated by experienced surgeons, the rate of primary anastomosis is suggested to be higher [12, 13]. Additionally, oncological outcomes are suggested to be equal between L-TME and R-TME [14].

Although oncological outcomes in patients operated by experienced surgeons might be equal, discussion remains regarding oncological results during the learning curve, especially since a previous study showed high local recurrence rates during the learning curve of R-TME [15], and two others showed high local recurrence rates during the learning curve of transanal TME (TaTME) [16, 17]. Although it is suggested that length of the learning curve of R-TME is around 40 cases [18–21], only few studies discuss oncological outcomes during the learning curve of R-TME, with varying results [19, 22]. Since multicentre data regarding local recurrence of patients operated with R-TME during the learning curve is lacking, this study aims to evaluate the 3-year local recurrence rates in the initial cases of R-TME surgeons in four large Dutch centres. It was hypothesized that the implementation phase of R-TME would be safe regarding oncological outcomes.

## Materials and methods

A retrospective multicentre cohort study was performed in four large Dutch hospitals. A protocol, which was not registered, regarding the design, methods, and statistical analysis, was composed prior to the initiation of the study. This study was reported in accordance with the STROBE guidelines [23]. The initial R-TME procedures since introduction of the technique in each centre were included, and a split group analysis was done for the initial 10 cases per surgeon (implementation phase), 11–40 cases per surgeon (learning phase), and 41 cases and onwards per surgeon (experienced phase), as the learning curve of R-TME is suggested to be around 40 procedures, and a recent study assessing oncological outcomes during the implementation of TaTME performed the same split group analyses for the initial 10, 11–40, and 41 cases and onwards [16, 18–20].

### Patients

Patients were eligible for inclusion if they (1) needed total mesorectal excision, (2) were diagnosed with rectal cancer according to the definition as proposed by D’Souza et al. [24], (3) were 18 years or older, and (4) were operated in an elective setting with (5) curative intent and (6) if the performing surgeon had performed > 20 robot cases during the inclusion period. There were no predefined exclusion criteria. Pre-operative work-up, treatment, and follow-up were according to the latest Dutch national guideline for rectal cancer [25]. In short this consisted of colonoscopy with pathological biopsy of the tumour, magnetic resonance imaging (MRI) of the rectum, imaging of the thorax and liver by either X-ray and ultrasound or CT for both. Neoadjuvant therapy in the form of chemoradiation was offered in case of threatened mesorectal fascia (MRF) or cN2 disease. In case of cT3 disease with more than 5 mm extramural invasion or cN1 disease, short-course radiotherapy was offered. Final treatment decisions were made in a multidisciplinary team meeting. Follow-up consisted of 6 monthly CEA and imaging of chest and liver during the first 2 years, and thereafter yearly up to 5 years.

### Outcomes

The primary outcome was the comparison of (multifocal) local recurrence at 3 years of follow-up between the initial 10 R-TME cases (implementation phase), cases 11–40 (learning phase), and case 41 and onwards (experienced phase) per surgeon. Secondary outcomes include comparison of overall survival, disease-free survival, and systemic recurrence at 3 years follow-up between the implementation phase, learning phase, and experienced phase.

### Data capturing

All hospitals provided their local data of the obligatory Dutch Colorectal audit (DCRA), including the unique patient number. After pseudonymization, missing and incomplete data was added to the database by accessing the local hospitals’ electronical medical record (EMR). In addition, local recurrence, systemic recurrence, and survival data were added using the local hospitals’ EMR. Informed consent was deemed unnecessary according to the Dutch Medical Treatment Agreement Act. The regional medical ethical committee and local ethical committees of all hospitals gave approval for the study (MEC-U, AW20.002, W19.096).

Baseline characteristics included age, sex, BMI, ASA classification, distance of the tumour to the anorectal junction (ARJ) on MRI, pre-operative mesorectal fascia involvement, neoadjuvant therapy, type of procedure performed, sequential case performed per centre, clinical and pathological TNM classification, histological tumour type, positive circumferential resection margin rate and quality of the TME according to Quirke [26]. Positive CRM was defined as any tumour tissue at a distance of ≤ 1 mm from the circumferential margin. Additionally, 30-day morbidity, mortality, reintervention rate, readmission rate, and anastomotic leakage rate were registered. Thirty-day morbidity was classified according to Clavien-Dindo [27]. Major morbidity was defined as Clavien-Dindo grade III or higher. Anastomotic leakage within 30 days was registered and classified according to the definition of the International Study Group of Rectal Cancer [28]. Overall survival was defined as being alive at 3 years of follow-up. Disease-free survival was defined as being alive without recurrent disease at 3 years of follow-up. Systemic recurrence was defined as any distant metastasis, either pathologically proven or as a lesion suspect for metastasis on imaging that showed growth on consecutive imaging. Local recurrence was defined as tumour deposit located in the pelvic cavity, with pathological proven adenocarcinoma, or growth on consecutive imaging if histopathological confirmation was absent. Multifocal local recurrence was defined as two or more separate deposits of recurrence in the pelvis. Location of local recurrence was classified according to the classification by Georgiou et al. [29].

### Robot-assisted training programme

All four centres started with R-TME after an e-learning, animal surgery, and proctoring of five procedures by an experienced R-TME surgeon as part of the training programme of Intuitive Surgical. The proctored cases were included in the implementation phase (cases 1–10). All surgeons adopting the R-TME technique in the four different centres had extensive experience with L-TME before starting with R-TME, with more than 200 L-TME and more than 100 open TME procedures performed per surgeon. Centres started with the technique between 2011 and 2016. In centre the A cases were operated using the DaVinci Xi, performed by one dedicated surgeon. Centre B and C used the DaVinci Si, and in both centres, two dedicated surgeons and a dedicated team of OR nurses performed the procedures. Centre D used the DaVinci Xi, performed by two dedicated surgeons.

### Statistical analysis

Analyses were conducted using R (version 3.6.1). Categorical and binary variables were compared using the *X*^2^ test. Continuous variables were compared using the independent *T*-test or the Mann–Whitney test, depending on the distribution. Survival curves were plotted in Kaplan-Meijer graphs. Comparisons were made between the initial 10 cases (implementation phase), cases 11–40 (learning phase), and case 41 and onwards (experienced phase) per surgeon. Finally, a multivariable Cox regression analysis, using backward regression, was performed for local recurrence at 3 years of follow-up to evaluate the independent effect of case load per surgeon. Confounding factors taken into account were sex (male/female), BMI (< 25/25–30/ > 30), distance of the tumour from the ARJ (≤ 3 cm/ > 3 cm), mesorectal fascia involvement on pre-operative MRI (yes/no), neoadjuvant therapy (none/radiotherapy/chemoradiation), pathological T stage (0–2/3–4), pathological N stage (0/1–2), pathological M stage (0/1), pathological CRM (≤ 1 mm, > 1 mm), TME quality (incomplete/complete or nearly complete), and pelvic sepsis (no/yes).

## Results

**Fig. 1:**
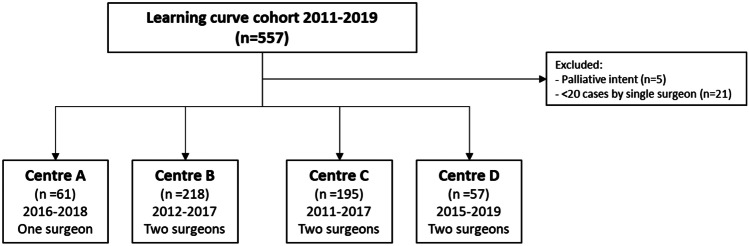
Flow diagram of included patients

A total of 557 patients were identified in the selected hospitals as receiving an R-TME, after exclusion of 5 patients that were treated with palliative intent, and 21 cases that were treated by surgeons that performed < 20 patients. This resulted in 531 patients, with 70 patients in the implementation phase (cases 1–10 per surgeon), 189 patients in the learning phase (cases 11–40 per surgeon), and 272 patients in the experienced phase (case 41 and onwards) (Fig. 1, Table 1).

**Table 1:** Baseline characteristics

		Total (531)	Cases 1–10 (70)	Cases 11–40 (189)	Case > 40 (272)	*p*-value
Age (mean, SD)		67 (10.4)	66 (9.2)	66 (10.6)	67 (10.6)	0.57
BMI (mean, SD)		26 (4.0)	25 (4.4)	26 (3.9)	26 (4.0)	0.65
Sex (*n*, %)	Male	333 (62.7)	43 (61.4)	120 (63.5)	170 (62.5)	0.95
	Female	198 (37.3)	27 (38.6)	69 (36.5)	102 (37.5)	
ASA classification (*n*, %)	I	89 (16.8)	12 (17.1)	31 (16.4)	46 (16.9)	0.91
	II	334 (62.9)	41 (58.6)	122 (64.6)	171 (62.9)	
	III	108 (20.3)	17 (24.3)	36 (19.0)	55 (20.2)	
	IV	0 (0.0)	0 (0.0)	0 (0.0)	0 (0.0)	
History of abdominal surgery (*n*, %)		160 (30.1)	24 (34.3)	60 (31.7)	76 (27.9)	0.49
Distance tumour from ARJ (median [IQR])		5 [2, 7]	5 [1, 7]	5 [1, 7]	6 [2, 7]	0.30
Mesorectal fascia involvement (*n*, %)	No	336 (63.3)	36 (51.4)	124 (65.6)	176 (64.7)	< 0.001
	Yes	162 (30.5)	21 (30.0)	57 (30.2)	84 (30.9)	
	Missing	33 (6.2)	13 (18.6)	8 (4.2)	12 (4.4)	
cT (*n*, %)	1	14 (2.6)	2 (2.9)	6 (3.2)	6 (2.2)	0.001
	2	144 (27.1)	10 (14.3)	52 (27.5)	82 (30.1)	
	3	291 (54.8)	44 (62.9)	106 (56.1)	141 (51.8)	
	4	71 (13.4)	8 (11.4)	21 (11.1)	42 (15.4)	
	Missing	11 (2.1)	6 (8.6)	4 (2.1)	1 (0.4)	
cN (*n*, %)	0	217 (40.9)	26 (37.1)	80 (42.3)	111 (40.8)	0.51
	1	167 (31.5)	22 (31.4)	57 (30.2)	88 (32.4)	
	2	137 (25.8)	19 (27.1)	47 (24.9)	71 (26.1)	
	Missing	10 (1.9)	3 (4.3)	5 (2.6)	2 (0.7)	
cM (*n*, %)	0	484 (91.1)	61 (87.1)	174 (92.1)	249 (91.5)	0.47
	1	33 (6.2)	7 (10.0)	12 (6.3)	14 (5.1)	
	Missing	14 (2.6)	2 (2.9)	3 (1.6)	9 (3.3)	
Neoadjuvant therapy (*n*, %)	None	155 (29.2)	14 (20.0)	53 (28.0)	88 (32.4)	0.34
	Chemoradiation	194 (36.5)	26 (37.1)	68 (36.0)	100 (36.8)	
	Radiotherapy	181 (34.1)	30 (42.9)	68 (36.0)	83 (30.5)	
		1 (0.2)	0 (0.0)	0 (0.0)	1 (0.4)	

*SD* standard deviation, *IQR* interquartile range, *BMI* body mass index, *ASA classification* American Society of Anesthesiologists, *ARJ* anorectal junction, *cTNM* clinical TNM stage

### Baseline characteristics

Patients had a mean age of 67 years (SD 10.4), with a mean BMI of 26 (SD 4.0). The majority of patients (62.7%) were male, and most were classified as ASA II (62.9%). The median distance of the tumour to the ARJ was 5 cm (IQR 3–8), a cT4 tumour was present in 13.4% of the patients, and the majority of patients received neoadjuvant therapy, either by chemoradiation (36.5%) or radiotherapy alone (34.1%). In the implementation phase a lower percentage of MRF involvement was observed with a higher percentage of missing data. In the same group, a lower percentage of cT2 tumours was observed (Table 1).

Regarding the postoperative outcomes, 33.9% of the patients underwent an APR, 55.8% underwent a LAR with the construction of an anastomosis, and 10.4% underwent a LAR with the construction of an ending colostomy. The TME specimen was incomplete in 3.4% of patients, while a positive CRM was present in 5.8% of the patients (Table 2). Significantly more patients had a metastasis in the group of the implementation phase.

**Table 2:** Short-term outcomes

		Total (531)	Cases 1–10 (70)	Cases 11–40 (189)	Case > 40 (272)	*p*-value
Procedure (*n*, %)	APR	180 (33.9)	24 (34.3)	66 (34.9)	90 (33.1)	0.52
	LAR + colostomy	55 (10.4)	9 (12.9)	23 (12.2)	23 (8.5)	
	LAR + anastomosis	106 (20.0)	9 (12.9)	38 (20.1)	59 (21.7)	
	LAR + anastomosis + dev ileostomy	190 (35.8)	28 (40.0)	62 (32.8)	100 (36.8)	
pT (*n*, %)	0	52 (9.8)	8 (11.4)	17 (9.0)	27 (9.9)	1.00
	1	51 (9.6)	6 (8.6)	20 (10.6)	25 (9.2)	
	2	179 (33.7)	23 (32.9)	67 (35.4)	89 (32.7)	
	3	234 (44.1)	31 (44.3)	79 (41.8)	124 (45.6)	
	4	13 (2.4)	2 (2.9)	5 (2.6)	6 (2.2)	
	Missing	2 (0.4)	0 (0.0)	1 (0.5)	1 (0.4)	
pN (*n*, %)	0	349 (65.7)	53 (75.7)	128 (67.7)	168 (61.8)	0.08
	1	140 (26.4)	11 (15.7)	49 (25.9)	80 (29.4)	
	2	40 (7.5)	6 (8.6)	10 (5.3)	24 (8.8)	
	Missing	2 (0.4)	0 (0.0)	2 (1.1)	0 (0.0)	
pM (*n*, %)	0	496 (93.4)	57 (81.4)	180 (95.2)	259 (95.2)	< 0.001
	1	26 (4.9)	6 (8.6)	7 (3.7)	13 (4.8)	
	Missing	9 (1.7)	7 (10.0)	2 (1.1)	0 (0.0)	
TME quality (*n*, %)	Incomplete	18 (3.4)	2 (2.9)	6 (3.2)	10 (3.7)	0.91
	Nearly complete	91 (17.1)	9 (12.9)	37 (19.6)	45 (16.5)	
	Complete	417 (78.5)	58 (82.9)	145 (76.7)	214 (78.7)	
	Missing	5 (1.0)	1 (1.4)	1 (0.5)	3 (2.1)	
CRM ≤ 1 mm (*n*, %)		31 (5.8)	5 (7.1)	8 (4.2)	18 (6.6)	0.50
Morbidity (*n*, %)	None	281 (52.9)	46 (66.7)	99 (52.4)	135 (49.5)	0.08
	Minor (CD 1–2)	159 (29.9)	11 (15.9)	60 (31.7)	88 (32.4)	
	Major (CD ≥ 3)	91 (17.2)	12 (17.4)	30 (15.9)	49 (18.1)	
	Abscess	33 (6.2)	4 (5.7)	15 (7.9)	14 (5.1)	0.47
	Anastomotic leakage	37 (12.4)	5 (13.2)	12 (11.9)	20 (12.5)	0.98
	Ileus	84 (15.8)	7 (10.1)	23 (12.2)	54 (19.9)	0.03
Reintervention (*n*, %)		74 (13.9)	9 (12.9)	26 (13.8)	39 (14.3)	0.92
Readmission (*n*, %)		92 (17.3)	10 (14.3)	34 (18.0)	48 (17.6)	0.77
LOS (median [IQR])		6 [4, 9]	7 [4, 8]	6 [4, 7]	6 [3, 10]	0.23

*APR* abdominoperineal resection, *LAR* low anterior resection, *pTNM* pathological TNM stage, *TME* total mesorectal excision, *CRM* circumferential resection margin, *CD* Clavien-Dindo, *LOS* length of stay

### Local recurrence

Local recurrence at 3 years was present in 25 cases (5.5%) in the total group. This was equally distributed between the implementation phase, learning phase, and experienced phase (4.9% versus 6.6% versus 5.0%, *p* = 0.85). Multifocal local recurrence was seen in 0% versus 37.5% versus 12.5% of the cases of local recurrence for respectively the implementation, learning, and experienced phase (Table 3). Univariable Cox regression analysis showed that mesorectal fascia involvement, pT stage 3–4, pN stage 1–2, positive circumferential resection margin, and incomplete TME margin were associated with local recurrence. In the multivariable analysis, only mesorectal fascia involvement (OR 2.73 [95% CI: 1.21, 6.14]) and pN stage 1–2 (OR 4.27 [95% CI: 1.84, 9.93]) remained. The implementation phase, learning phase, and experienced phase were not associated with difference in local recurrence (Table 5).

**Table 3:** Long-term oncological outcomes

	Level	Total (531)	Cases 1–10 (70)	Cases 11–40 (189)	Case > 40 (272)	*p*-value
Follow-up time (median [IQR])		32 [19, 50]	43 [30, 61]	26 [19, 58]	33 [18, 44]	< 0.001
3-year overall survival (*n*, %)		473 (88.3)	62 (89.5)	169 (87.7)	242 (88.5)	0.97
3-year disease-free survival (*n*, %)		405 (73.1)	53 (73.1)	145 (74.1)	207 (72.1)	0.95
3-year local recurrence (*n*, %)		25 (5.5)	3 (4.9)	10 (6.6)	12 (5.0)	0.85
	Multifocal	5 (16.7)	0 (0.0)	3 (37.5)	2 (12.5)	0.14
3-year systemic recurrence (*n*, %)		93 (21.0)	17 (25.8)	31 (18.4)	45 (21.5)	0.37

*IQR* interquartile range

Out of the 25 cases of local recurrence, three cases occurred during the implementation phase and 10 cases occurred during the learning phase. Five cases of local recurrence were solely local recurrences without additional systemic recurrences, two cases developed a systemic recurrence after an initial local recurrence, and 9 cases of local recurrence were accompanied with a systemic recurrence. In all but four patients, local recurrence was treated with palliative intent, either due to age and co-morbidities or due to progression of the disease (Tables 4 and 5).

**Table 4:** Cases of local recurrence during the initial 40 cases per surgeon

Surgeon	Case	Age	Sex	Tumour height (MRI)	Neoadjuvant therapy	Surgery	Pelvic sepsis	pTNM	Tumour differentiation	CRM margin	TME quality	Follow-up	Treatment	Last follow-up
A	37^th^	56	F	10 cm	-	LAR	Yes	pT3N0M0	W/M	5 mm	Complete	288 days LR + SR (lung, peritoneal, bone)	Palliative	455 days alive with disease
B_1_	23^th^	70	M	4 cm	Radiotherapy	LAR + colostomy	No	pT3N2M0	W/M	> 10 mm	Complete	134 days LR + SR (lung, bone)	Palliative	168 days alive with disease
B_1_	33^th^	50	F	3 cm	Chemoradiation	LAR + deviating ileostomy	No	pT3N1M0	W/M	0 mm	Incomplete	174 days LR, 216 days SR (lung + liver)	Curative	610 days alive with disease
B_2_	3^th^	61	M	0 cm	Chemoradiation	APR	No	pT3N2M0	W/M	1 mm	Unknown	216 days LR + SR (lung, liver)	Palliative	539 days alive with disease
B_2_	24^th^	71	F	2 cm	-	APR	No	pT3N1M0	Poor	0 mm	Complete	243 days LR + SR (liver, bone)	Palliative	608 days with disease
B_2_	30^th^	74	M	3 cm	-	APR	No	pT2N0M0	W/M	> 10 mm	Complete	780 days LR, 1304 days SR (liver, lung)	Palliative	1941 days deceased
C_1_	18^th^	80	F	8 cm	Radiotherapy	LAR + colostomy	No	pT3N2M0	Unknown	> 10 mm	Complete	605 days LR + SR (lung)	Palliative	737 days alive with disease
C_2_	6^th^	73	M	1 cm	Chemoradiation	APR	No	pT2N1M0	W/M	4 mm	Complete	711 days LR + SR (lung, peritoneal)	Palliative	1415 days deceased
C_2_	23^th^	63	F	6 cm	Radiotherapy	LAR + deviating ileostomy	No	pT3N1M0	W/M	> 10 mm	Complete	575 days LR	Curative	1301 days alive without disease
C_2_	35^th^	75	M	6 cm	Radiotherapy	LAR + deviating ileostomy	No	pT2N0M0	W/M	8 mm	Nearly complete	314 days LR	Palliative	636 days alive with disease
C_2_	38^th^	74	M	8 cm	Chemoradiation	LAR	No	pT3N1M0	W/M	2 mm	Complete	700 days LR	Curative	960 days with disease
D_1_	4^th^	81	F	2 cm	Chemoradiation	APR	Yes	pT3N0M0	W/M	> 10 mm, DRM +	Complete	553 days LR	Curative	1376 alive without disease
D_1_	24^th^	66	M	0 cm	Chemoradiation	APR	No	pT2N0M0	W/M	> 10 mm	Complete	311 days LR	Palliative	553 alive with disease

*MRI* magnetic resonance imaging, *pTNM* pathological TNM classification, *W/M* well/moderate, *CRM* circumferential resection margin, *DRM* distal resection margin, *TME*, total mesorectal excision, *F* female, *M* male, *LAR* low anterior resection, *APR* abdominoperineal resection, *LR* local recurrence, *SR* systemic recurrence

**Table 5:** Univariable and multivariable Cox regression analysis for local recurrence

Variable		Univariable analysis		Multivariable analysis	
		OR (95% CI)	*p*	OR (95% CI)	*p*
Experience	Cases 1–10	Reference		NA	
	Cases 11–40	1.98 [0.08, 3.02]	0.45		
	Case > 40	0.87 [0.27, 4.91]	0.85		
Sex	Male	Reference		NA	
	Female	1.52 [0.69, 3.43]	0.29		
BMI	< 25	Reference		NA	
	25–30	0.42 [0.15, 1.20]	0.11		
	> 30	2.06 [0.84, 5.04]	0.11		
Distance to ARJ	> 3 cm	Reference		NA	
	≤ 3 cm	1.81 [0.82, 3.99]	0.14		
Mesorectal fascia involvement	MRF-	Reference		Reference	
	MRF +	3.09 [1.33, 7.15]	0.01	2.73 [1.21, 6.14]	0.02
Neoadjuvant therapy	None	Reference		NA	
	Radiotherapy	2.68 [0.74, 9.73]	0.13		
	Chemoradiation	3.37 [0.95, 11.93]	0.06		
pT stage	0–2	Reference		NA	
	3–4	4.04 [1.61, 10.11]	0.002		
pN stage	0	Reference			
	1–2	4.51 [1.94, 10.44]	< 0.001	4.27 [1.84, 9.93]	< 0.001
pM status	0	Reference		NA	
	1	2.38 [0.71, 7.96]	0.16		
CRM	> 1 mm	Reference			
	≤ 1 mm	4.76 [1.79, 12.69]	0.002	NA	
TME quality	Complete/nearly complete	Reference			
	Incomplete	3.72 [1.28, 10.85]	0.016	2.40 [0.42, 7.14]	0.12
Pelvic sepsis	No	Reference		NA	
	Yes	1.41 [0.48, 4.11]	0.53		

*OR* odds ratio, *CI* confidence interval, *NA* not applicable, *ARJ* anorectal junction, *MRF* mesorectal fascia, *pM* clinical metastasis stage, *pT* pathological tumour stage, *pN* pathological nodal stage, *CRM* circumferential resection margin, *TME* total mesorectal excision

### Survival and systemic recurrence

**Fig. 2:**
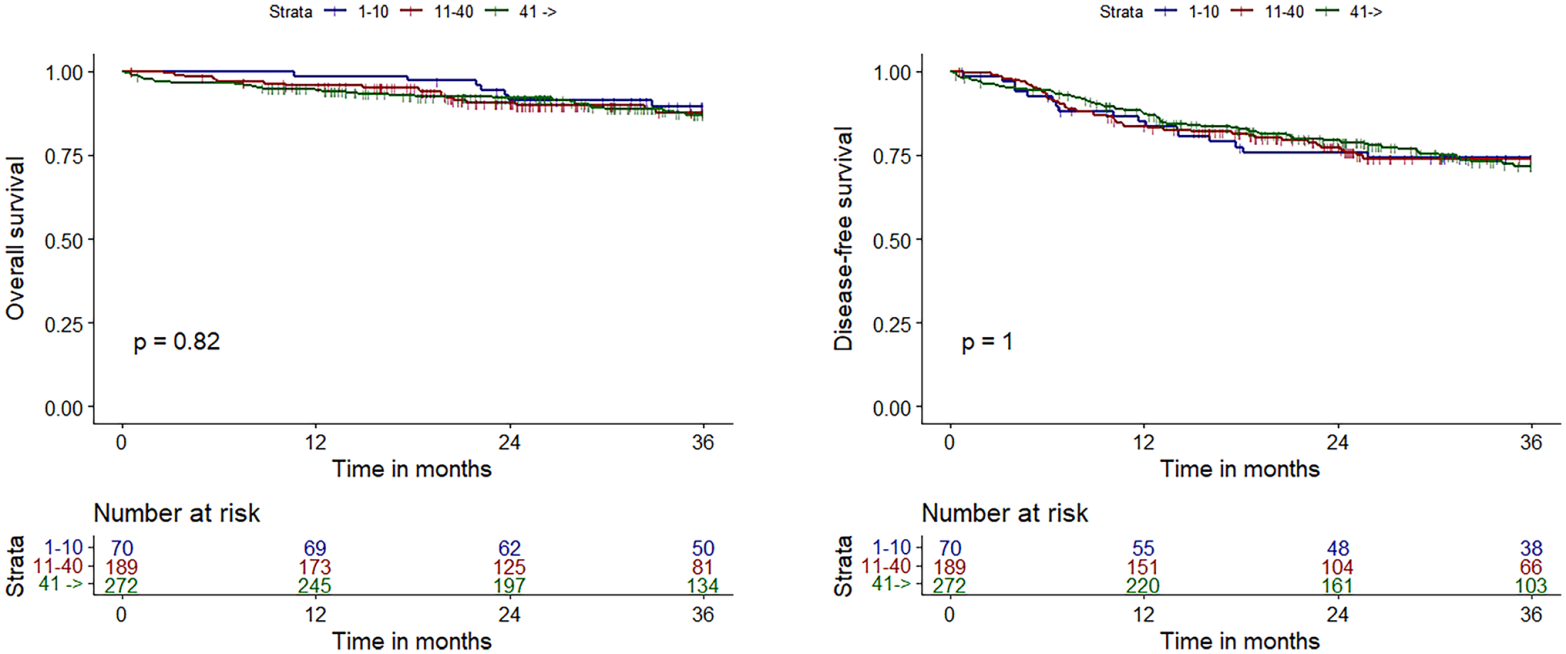
Overall survival and disease-free survival at 3 years follow-up during the initial 10 cases, subsequent 30 cases, and the final cases performed by the seven robot-assisted surgeons

Median follow-up time was 32 months (IQR: 19–50). Overall survival at 3 years of follow-up was 89.1%, disease-free survival was 76.8%, and systemic recurrence was 20.2%. No significant differences between the different phases were found (Fig. 2 and Table 3).

## Discussion

This study aimed to describe oncological outcomes in four Dutch R-TME centres during the period of introduction of the R-TME technique, and shows a local recurrence rate of 4.9% at 3 years of follow-up for the initial 10 cases per surgeon and a local recurrence rate of 6.6% for cases 11–40 per surgeon. Implementation phase, learning phase, or experienced phase was not associated with a higher rate of local recurrence.

Local recurrence was adequate with 4.9%, 6.6%, and 5.0% at 3 years of follow-up. This is in line with results of large randomized controlled trials comparing open with L-TME, showing local recurrence rates of around 5% [3–6]. In our study, only patients with a rectal tumour according to the new definition by D’Souza et al. [24] were included, and patients with cT4 tumours or mesorectal fascia involvement on pre-operative MRI were not excluded. Compared to the aforementioned large randomized controlled trials, this could have led to the inclusion of more difficult tumours, as cT4 tumours, pre-operative mesorectal fascia involvement, and lower tumours are associated with a higher risk of local recurrence [30]. Furthermore, patients operated during the implementation phase had a significantly higher rate of pM1. Nevertheless, this did not result in worse oncological outcomes.

Other studies reporting on local recurrence rates in R-TME centres during implementation of the technique have described low local recurrence rates as well [31–35]. However, these studies are mostly small studies, subject to significant bias, with short follow-up times in a relatively young and healthy population. In contrast, a Dutch single-centre study regarding the initial 77 cases of R-TME showed a local recurrence rate of 9.5% at 2 years of follow-up [15]. Additionally, the authors reported a positive CRM rate of 10.4%, suggesting inadequate technical dissection, as an effect of the learning curve that had not yet been fulfilled. These results are not in line with the results of our study. Perhaps this could be explained by difference in experience of the surgeon with L-TME, as all surgeons in the present study had profound experience with L-TME. L-TME and R-TME both use a top-down approach; therefore, experience with L-TME might influence outcomes of R-TME during the initial cases. More recently, a study reporting on the implementation of R-TME in a large centre with surgeons having profound experience with L-TME showed a local recurrence rate of 4.0% at 2 years of follow-up with a median follow-up of 28 months [36]. This supports our suggestion that the learning curve of R-TME does not lead to additional local recurrences if performed by surgeons having experience with L-TME.

This is in contrast to the learning curve of TaTME that is suggested to be associated with local recurrence rates of up to 10%, with multifocal recurrence rates of up to 67% during the learning curve [16, 17, 37]. We suggest that this might be due to the fact that TaTME uses a bottom-up approach, in which anatomical landmarks are different compared with the top-down approach of open TME, L-TME, and R-TME. Additionally, the use of a purse string in the TaTME technique is suggested to be an explanation for the difference in (multifocal) local recurrence between the techniques [37]. Although high rates of local recurrence have been observed in these studies, low rates of local recurrence have been shown as well, especially in studies reporting on oncological outcomes after obtaining the learning curve of TaTME [16, 38–40]. Perhaps experience with the technique is more important than the technique itself [41]. Finally, reports on additional morbidity are not limited to TaTME only. During the beginning of the adaptation of laparoscopic colorectal surgery, reports demonstrated local recurrence rates of 10.5% and the occurrence of port-site metastasis during the learning curve [42, 43].

Although our results suggest that R-TME performed by experienced L-TME surgeons does not lead to additional local recurrence during the initial cases after introduction of R-TME, certain limitations should be taken into account regarding the results of this study. First, this is a retrospective study, which bares the risk of selection bias. More importantly, this might be even more apparent as patients are especially selected during the beginning of the implementation of a new technique. Mostly patients with ‘easy’ tumours are selected during the initial phase of implementation, while patients with more ‘difficult’ tumours are operated using the new technique after a certain degree of experience has been established. Although a lower rate of mesorectal fascia involvement and cT2 rate was observed in the group containing the initial 10 cases per surgeon, it is unlikely that this affected outcomes, as pathological T stage and positive CRM were comparable. Furthermore, we did not exclude patients with cT4 tumours or stage IV disease and we only included patients with a rectal tumour according to the new definition by D’Souza et al. [24]. This could potentially have led to relatively more low rectal tumours, and therefore more APRs, as recto-sigmoidal tumours have been excluded due to this definition. Nevertheless, baseline characteristics of our cohort do not reveal such selection bias after comparison with national data [44]. Secondly, although this study aims to include patients operated during the learning curve, a clear definition of the learning curve is as of yet lacking. Since earlier reports on the learning curve in R-TME suggest length of the learning curve to be between 20 and 75 patients, and a recent study aimed at assessing oncological outcomes during the implementation of TaTME included the first 40 patients who underwent TaTME, we used the latter inclusion criteria for comparability. Thirdly, this study reports on oncological outcomes during the introduction of the technique of only four centres. Preferably more centres would have been included, resulting in greater external validity. In addition, the contributing centres differed regarding the starting year of the technique, number of annually operated patients and patients included, which might have affected outcomes. However, despite these differences, local recurrence remained within clinically safe margins. Fourth, this is a non-comparative study. Therefore, additional prospective studies comparing the learning curve of the minimal invasive techniques should be performed. Finally, we did not perform a risk-adjusted cumulative sum analysis (RA-CUSUM). Although this might be the preferred method for evaluating the learning curve, this method is not favourable for outcomes with a low incidence such as local recurrence.

Concluding, the use of R-TME during the implementation of the technique, performed by experienced L-TME surgeons, is safe with regard to oncological outcomes, and more specifically local recurrence. R-TME might be a safe alternative besides L-TME and TaTME, as it does not lead to additional local recurrence during the initial cases. Nevertheless, comparative prospective studies are necessary to directly compare results of L-TME, R-TME, and TaTME during the learning curve.
